# Prehaustoria of root hemiparasites *Rhinanthus minor* and *Odontites vernus* (Orobanchaceae) produce lignin-rich interfacial deposits closely resembling those of attached haustoria

**DOI:** 10.1093/aob/mcaf149

**Published:** 2025-07-19

**Authors:** Anna Pielach, Gordon G Allison, Olivier Leroux, Zoë A Popper

**Affiliations:** Botany and Plant Science and Ryan Institute for Environmental, Marine and Energy Research, School of Natural Sciences, University of Galway, Newcastle Road, Galway, H91 TK33, Ireland; Department of Biological and Environmental Sciences, University of Gothenburg, Gothenburg 40530, Sweden; Department of Life Sciences, Aberystwyth University, Aberystwyth, SY23 3DA, UK; Department of Biology, Ghent University, K.L. Ledeganckstraat 35, 9000 Gent, Belgium; Botany and Plant Science and Ryan Institute for Environmental, Marine and Energy Research, School of Natural Sciences, University of Galway, Newcastle Road, Galway, H91 TK33, Ireland

**Keywords:** Lignin, haustorium, prehaustorium, hemiparasite, cell wall, *Rhinanthus minor*, *Odontites vernus*

## Abstract

**Background and Aims:**

Lignin and other phenolics are commonly observed at the interfaces between the haustoria of parasitic plants and tissues of their hosts. As known plant defence compounds, their accumulation at haustorial interfaces has been ascribed to mechanical and chemical resistance of host tissues. Although the possibility that the interfacial lignin deposits may have a parasitic origin has not previously been addressed, the fact that certain parasitic plants, including *Rhinanthus* and *Odontites*, can form haustoria in the absence of hosts gives us a tool that can be used to help answer this question.

**Methods:**

We compared the interfaces of haustoria of root hemiparasites yellow rattle *Rhinanthus minor* and red bartsia *Odontites vernus* (Orobanchaceae) attached to hosts bulbous oat-grass *Arrhenatherum elatius* ssp. *bulbosum* and perennial rye grass *Lolium perenne* (Poaceae) with the contact surfaces of non-infective prehaustoria attached to a pot surface. We performed histochemistry, immunocytochemistry and Raman spectroscopy to characterize the architecture of contact deposits formed by both.

**Key Results:**

Lignolic deposits, which we will term lignin-rich interfacial deposits (LIDs), were found at the interfaces between haustoria and compatible hosts as well as at the pot-appressed facets of prehaustoria. In both cases the deposits were determined as lignin by histology and Raman spectroscopy. Xyloglucan and arabinogalactan protein glycan epitopes were also detected while mixed-linkage glucan, xylans and pectin were not. We demonstrate that prehaustoria can produce lignolic interfacial deposits of high structural similarity to those of haustorium-host interfaces.

**Conclusions:**

LIDs at haustorium–host interfaces may at least partly be attributed to the parasite and benefit the establishment and functioning of the haustorium. A reinterpretation of the origin and role of interfacial lignin in parasitic plant–host interactions may therefore be necessary.

## INTRODUCTION

Lignin is a hydrophobic, racemic polyphenol produced by plants. It is a constitutive component of xylem vessel and fibre cell walls, where it typically encrusts a scaffold rich in cellulose and xylem with a low pectin content (Albersheim *et al*., 2010). Due to its recalcitrant properties lignin production is a basal defence mechanism (Chezem *et al*., 2017) and lignin-like compounds are often deposited in response to pathogen infections and wounding (Nicholson and Hammerschmidt, 1992; Hückelhoven, 2007; Moura *et al*., 2010; Cabane *et al*., 2012; Sattler and Funnell-Harris, 2013; Cesarino, 2019). Induced lignification increases mechanical and biochemical resistance via incrustation of previously non-lignified walls and increased lignification of already lignified walls (Hawkins and Boudet, 2003; Eynck *et al*., 2009 , 2012; Kavuluko *et al*., 2021; Jhu *et al*., 2022; Mutinda *et al*., 2023), occlusion of damaged or infected vessels (Hawkins and Boudet, 2003), changes in monolignol composition (Hawkins and Boudet, 2003; Menden *et al*., 2007), intracellular accumulation coupled to hypersensitive response and cell death (Beardmore *et al*., 1983; Menden *et al*., 2007; Lee *et al*., 2019) and linking of constituent low-molecular-weight phenolics to each other or to carbohydrates, rendering walls recalcitrant to cell wall degrading enzymes (Albersheim *et al*., 2010). Although biochemical evidence for defence-induced lignification is plentiful, detailed localization studies showing precise topochemistry of polyphenolic reinforcement are rare (Frankenstein *et al*., 2005; Rehbein and Koch, 2011).

Polyphenolics have been also reported from the interfaces between haustoria of parasitic plants and their plant host tissues. Haustoria are specialized intrusion organs which use mechanical force coupled with enzymatic action to access solutes in host vasculature (Pérez-de-Luque, 2013; Kokla and Melnyk, 2018; Balios *et al*., 2025). Enzymes play a role not only in digesting the host walls but also remodelling the haustorial walls (Kokla and Melnyk, 2018; Leso *et al*., 2023; Balios *et al*., 2025; Barminga *et al*., 2025). Lignin also plays various integral roles in the development of haustoria, starting by their formation being triggered by host lignin-derived haustorium-inducing factors (HIFs) (Goyet *et al*., 2019). Furthermore, HIFs stimulate lignification of the parasite *Striga hermonthica* (Orobanchaceae) root tip during infection by upregulation of enzymes involved in lignin synthesis and polymerization (Cui *et al*., 2025). Moreover, lignification underpins the development of the haustorial secondary xylem bridge connecting the vasculatures of the parasite and the host (Heide-Jørgensen and Kuijt, 1995; Teixeira-Costa, 2021; Kirschner *et al*., 2023) and upregulation of genes related to lignin metabolism in the haustoria has been observed (Sun *et al*., 2018; Vogel *et al*., 2018; Brun *et al*., 2023; Leman *et al*., 2025). At haustorial attachment sites polyphenolics often accumulate along the contact surfaces between the haustorium and host tissues. This phenolic-enriched contact zone is typically interpreted as an encapsulation layer enforced by the host to physically block the haustorium (Visser *et al*., 1990; Labrousse *et al*., 2001; Zehhar *et al*., 2003; Cameron *et al*., 2006; Rümer *et al*., 2007; Yoshida and Shirasu, 2009; Mutuku *et al*., 2019; Somssich and Cesarino, 2022; Barminga *et al*., 2025). While descriptions such as ‘lignified cells’ are used in some studies (Somssich and Cesarino, 2022) the phenolic-rich features identified in encapsulation layers suggest a wide range of deposition mechanisms and their accumulative effect. These include lignin-encrusted host cell walls (Maiti *et al*., 1984; Hood *et al*., 1998; Pérez-de-Luque *et al*., 2005, 2007; Mutuku *et al*., 2019), osmiophilic substances filling the narrow space between the mature haustorium and host cells (Piehl, 1963; Musselman and Dickison, 1975), and dead host cells (Cameron *et al*., 2006; Rümer *et al*., 2007). While lignin deposition is known to aid resistance to parasitic plants (Jhu *et al*., 2023; Mutinda *et al*., 2023) the evidence for its contribution to resistance is often circumstantial. Nonetheless, interfacial lignin deposition appears to be conserved across various parasitic plant lineages (Ichihashi *et al*., 2018). Therefore, determining its role and deposition mechanism in plant host–parasite interactions is important as it underpins the broad-scale impacts of parasitism, namely its positive effects on biodiversity (Press and Phoenix, 2005) and those detrimental to crop yields (Parker, 2009, 2012).

Some authors have cautiously suggested that the parasite might also synthesize interfacial lignin to facilitate attachment to the host in *Pedicularis canadensis* (Orobanchaceae) (Piehl, 1963), *Rhinanthus minor* (Orobanchaceae) (Rümer *et al*., 2007) and *Santalum album* (Santalaceae) (Tennakoon and Cameron, 2006), sealing and prevention of water loss during solute abstraction in *R. minor* (Rümer *et al*., 2007), and strengthening of infective cells to prevent fungal infection in *Triphysaria* (Orobanchaceae) (Heide-Jørgensen and Kuijt, 1993) and *Buchnera hispida* (Orobanchaceae) (Neumann *et al*., 1999). Losner-Goshen *et al*. (1998) remarked that osmiophilic coating on the lateral haustorial surface of *Orobanche aegyptiaca* (Orobanchaceae) extended to regions not in direct contact with host tissues. Unfortunately, none of the above findings were further investigated or discussed, presumably because of technical difficulties. It is problematic to pinpoint the origin of substances sandwiched between the host and the parasite and so far it has been successfully demonstrated only for the shoot-parasitizing *Viscum minimum* (Santalaceae), in which an examination of various developmental stages showed that the interfacial cuticle-like adhesive secretion complex is produced by the haustorium (Heide-Jørgensen, 1989). This method is, however, harder to apply to root parasites as the haustoria occur below the soil surface, making it challenging to follow successive developmental stages and collect samples at high temporal resolution. Although soil-free culture protocols have been developed for some parasites, e.g. *Triphysaria* (Tomilov *et al*., 2004), these require specialized, sterile conditions and high maintenance, and are difficult to apply to non-model taxa of high ecological importance, such as *R. minor*. Furthermore, unlike in the case of stem-parasitizing *Cuscuta* (Convolvulaceae) species, the haustoria of root hemiparasites do not tend to arise in a continuous developmental sequence along the root but are randomly distributed. Finally, the very tight physical contact between the host and parasite may obscure determination of the organism responsible for secreting components present at the interface.

An alternative system is offered by non-infective haustoria that develop without physical contact with a host. These may be haustoria clasping around small stones (Piehl, 1963; A. Pielach pers. obs.), dead organic matter such as dead roots (Piehl, 1963) or dead twigs (Ren *et al*., 2010) as well as non-clasping haustoria that are mentioned in the literature under a variety of names. These have in some cases not been given any specific name, such as *Santalum album* haustoria attaching to the inner pot surface (Tennakoon and Cameron, 2006) or haustoria of *Aureolaria pedicularia* (Orobanchaceae) with a smooth distal surface and no signs of previous attachment, field-collected by Werth and Riopel (1979). ‘Metahaustoria’, a term coined by Weber (1976) and used by Kuijt (1977) and Attawi and Weber (1980), were defined as large (up to 3.5 mm wide) but non-parasitizing haustoria that presumably lost contact with the host root after initiation but developed many features characteristic of normal attached haustoria, e.g. a secondary xylem strand. However, in past decades a range of terms has been used, including: ‘pseudo-haustoria’ of *Cuscuta campestris* (Hong *et al*., 2011), hormone-induced and thigmotropic pseudo-haustoria of *Triphysaria* in culture (Tomilov *et al*., 2005), ‘spontaneous haustoria’ developed by cultured *Orthocarpus purpurascens* (Orobanchaceae) (Atsatt *et al*., 1978) and by *Pedicularis kansuensis* (Xiang *et al*., 2018), and *Agalinis purpurea* grown on agar (Riopel and Musselman, 1979; Steffens *et al*., 1982). Another, more broadly used term is ‘pre-haustoria’, e.g. those formed by *Cuscuta reflexa* on wooden sticks (Löffler *et al*., 1999) and on filter paper (Ramasubramanian *et al*., 1988) or by *Krameria lappacea* (Krameriaceae) collected in the wild (Brokamp *et al*., 2012). The term has recently been revisited by Furuta *et al*. (2021) who applied it to *in vitro*-induced early stage haustoria. In this study, we adopt the term ‘prehaustoria’ for the non-infective haustoria produced by herbaceous root hemiparasites *R. minor* (yellow rattle) and *Odontites vernus* (red bartsia) (Orobanchaceae) in the absence of a host.

*Rhinanthus minor* and *O. vernus* are common in grassland ecosystems of northern temperate regions and their general haustorial structure has been well researched (Cameron *et al*., 2006; Cameron and Seel, 2007; Rümer *et al*., 2007; Pielach *et al*., 2014). Notably, the haustoria of both species are rich in arabinogalactan proteins (AGPs) (Pielach *et al*., 2014), which play diverse roles in development and signalling (Lin *et al*., 2022; Ma and Johnson, 2023). *Rhinanthus minor* and *O. vernus* are facultative hemiparasites that can survive without a host, particularly when not subject to competition, and they often form haustoria that are not connected to a host root (Weber, 1976). *Rhinanthus minor* has a well-documented positive effect on the floral diversity of semi-natural grasslands attributed to shifts in competitive balance between its hosts and non-hosts (Davies *et al*., 1997; Pywell *et al*., 2004; Bullock and Pywell, 2005; Westbury *et al*., 2006). While Poaceae and Fabaceae include many compatible hosts, non-host resistance of non-leguminous forbs has been linked to lignin deposition at interfacial reinforcement sites which physically block haustorial development (Cameron *et al*., 2006; Cameron and Seel, 2007; Rümer *et al*., 2007). *Odontites vernus* is a species of roadsides, waste ground, arable land and disturbed pastures (Snogerup, 1982; Hirst *et al*., 2005; Albrecht *et al*., 2008). It has been demonstrated to form haustorial connections to various taxonomic host groups (Govier *et al*., 1967; Govier *et al*., 1968; Snogerup, 1982) and its ability to form successful haustorial connections with native expansive and non-native invasive species has recently been highlighted (Knotková *et al*., 2024). *Arrhenatherum elatius* ssp. *bulbosum* and *Lolium perenne* are associated with grassland habitat deterioration. *Arrhenatherum elatius* can become expansive or invasive where grazing or hay meadow management have been discontinued (Rodwell, 2000; Hinckley *et al*., 2022) while *L. perenne* is a commonly introduced component of amenity grasslands and grasslands intensively managed for fodder (Petkova *et al*., 2021; Wiewióra and Żurek, 2023; Prijović *et al*., 2025). Both species are therefore important targets for hemiparasitism-facilitated grassland restoration. Moreover, graminoid cell wall composition is very distinct from that of dicots. Low content of pectins and presence of xylans and mixed-linkage glucan (MLG) (Harris, 2005) facilitate clear immunocytochemical distinction from dicot parasite host cell walls which they come in close contact with at haustorial interfaces. To gain insights into the origin of lignolic interfacial substances, i.e. their deposition by the host versus the parasite, we compared the architecture of contact deposits in non-infective prehaustoria and haustoria of *R. minor* and *O. vernus* attached to roots of *A. elatius* ssp. *bulbosum* and *L. perenne*. We combined histology, immunocytochemistry and Raman spectroscopy to characterize the architecture of the deposits.

## MATERIALS AND METHODS

### Plant material and co-cultivation experiments

*Rhinanthus minor* and *Lolium perenne* (Poaceae) for wax embedding were grown from seeds purchased from Emorsgate Seeds in England (wildseed.co.uk). Seeds for all remaining species and experiments were collected in Ireland as follows. All resin-embedded haustoria of *R. minor* were obtained from seeds collected at Killanin Esker, Roscahill, Co. Galway (53°22′56.20″N, 9°12′00.00″W). All *Odontites vernus* seeds were collected from a road verge in Galway city (53°17′16.51″N, 9°04′13.12″W) and seeds of *Arrhenatherum elatius* ssp. *bulbosum* (Poaceae) were collected on the island of Inis Mór (53°07′53.25″N, 9°40′45.20″W). Seeds were collected during July and August and stored at room temperature in paper bags and germinated during the following autumn, winter and spring. *Rhinanthus minor* seed stratification was started no later than in February to avoid the negative effects of the short viability of its seeds.

Seeds of all species and for all experiments were sterilized by immersing in sodium hypochlorite in distilled water (dH_2_O) at 1.35 % w/v for 10 min and washed in dH_2_O. Seeds of all species except *L. perenne* were immediately placed in 9-cm Petri dishes lined with Whatman No. 1 filter paper soaked with an aqueous dilution of fungicide Bravo 500 (0.1 % v/v). The dishes were sealed with parafilm to prevent drying out. Thirty replicate dishes of 9-cm diameter were used per *R. minor* and *O. vernus* per each germination batch with a seed density of 25 per 9 cm diameter dish for *R. minor* and 50 seeds per dish for *O. vernus*. Half of the batches were cold-stratified at 4 °C and half under a 4 °C 12 h/0 °C 12 h regime. Germination was checked biweekly, and for both species and stratification regimes first germination was observed after 8 weeks. Germination continued to be observed for 4 weeks. Only seeds from which the radicle but not the cotyledons had emerged were planted, with hosts being gently placed in soil with the radicle facing down and the seed coat being visible on the surface.

Sterilized *L. perenne* seeds (Emorsgate) were sown in January in three trays of 38 cm (length) × 25 cm (width) × 5 cm (depth) filled with No. 1 John Innes young plant compost, ∼200 seeds (0,4 g) per tray. The trays were kept in a glass greenhouse with natural light and non-controlled temperature conditions. Germination started on day 6 and ∼20 % of seeds had germinated by day 21 (exact germination rate was not recorded) and no further germination was observed. Twenty germinating *R. minor* seeds (Emorsgate) were added to each tray 10 weeks after sowing the grass and thinned to four strong specimens per tray after 4 weeks. Haustoria were harvested and fixed when *R. minor* was flowering (7 weeks old). Additionally, *Rhinanthus* seedlings were planted in cavity inserts for seedling propagation (individual insert size 2 cm × 2.5 cm (bottom of cavity) to 3 cm × 3.5 cm (top of cavity) × 5 cm high). One plant per insert was grown without hosts in the greenhouse and harvested when flowering (10 weeks old). All watering was performed by filling drainage trays with tap water when the surface of the soil started to become dry (daily to once per week). More water was added until none was taken up and excess water was removed after 30 min to prevent waterlogging.

Sterilized dehusked caryopses of *A. elatius* were placed in ten Petri dishes at 20 per dish on a windowsill at room temperature (∼20 °C). The germinating seeds were planted in 40 round tapered plastic pots of 7.6 cm diameter at the top and 7.5 cm height (Stewart Garden). Plants were grown in a Binder KBW720 climate chamber programmed to a cycle of 22 °C and 16 h light at ∼100 μmol m^−2^ s^−1^ when measured at 10 cm below the light source and 15 cm above the top edge of the pots, followed by 8 h dark, 16 °C. Watering was performed as described above by filling saucers and removing excess water. We used a soil mix that could relatively easily be removed from the roots upon immersion in water while providing sufficient nutrition and water retention. It comprised 3 parts of well-mineralized, dark garden soil, 3 parts of kiln-dried paving sand (B&Q) and 1 part of heavy calcareous clay. Three germinating *R. minor* (Roscahill) seeds were added to ten pots with 7-week-old hosts and to a further ten with 11-week-old plants. Three germinating *O. vernus* seeds per pot were planted in ten pots with 9-week-old hosts. Parasite plants were thinned to one per pot 2 weeks from planting. Haustoria attached to host roots and metahaustoria attached to pot walls were harvested and fixed from flowering plants of *R. minor* and *O. vernus* that were 8–13 weeks old.

*Rhinanthus minor* (Roscahill) and *O. vernus* (Newcastle Road) were additionally planted with no hosts in trays 23 cm (length) × 17 cm (width) × 7 cm (depth), three trays per species, ten germinating seeds per tray, and grown in a Binder KBW720 climate chamber under the temperature, light and watering conditions described above. Parasite plants were thinned to five individuals per tray after 4 weeks. Metahaustoria appressed to tray surfaces were harvested from 15-week-old blooming plants and embedded in Steedman’s wax (*O. vernus*) and London Resin White (LR White) (*O. vernus* and *R. minor*) as specified below.

### Fixation and dehydration of haustoria

Haustoria were collected and fixed one pot or tray at a time to ensure prompt fixation. The roots were washed of soil manually by immersing in a container filled with water and gentle agitation and separation of roots. Haustoria were collected with fragments of parasite and host roots up to 3 mm long on each side of the haustorium. They were immediately fixed for 2 h either in 4 % v/v formaldehyde (FA, Sigma, F8775) in PEM buffer [50 mm piperazine-*N*,*N*′-*bis*(2-ethane-sulphonic acid) (PIPES) (Sigma, P-6757), 5 mm ethylene glycol *bis*(β-aminoethylether)-*N*,*N*,*N*′,*N*′-tetraacetic acid (EGTA) (Sigma, 03777), 5 mm MgSO_4_ (Sigma, M7506), pH 6.9] or 2 % v/v formaldehyde and 2.5 % glutaraldehyde (GA) (Agar Scientific, R1010) in PEM buffer. Ten haustoria or prehaustoria were placed in a 1.5-mL tube filled with the fixative at room temperature and moved to the refrigerator at 4 °C after 15 min. Primary fixation was followed by three 5-min washes in phosphate-buffered saline (PBS, Agar Scientific, P5493). Half of the FA + GA-fixed haustoria were post-fixed for 2 h in 1 % w/v osmium tetroxide (Agar Scientific, R1023) in PEM buffer in 1.5-ml Eppendorf tubes at 4 °C. Fixed and postfixed samples were washed three times for 5 min with PBS and dehydrated in a graded ethanol (EtOH, Lennox, CH314) series (30, 50, 70, 90 and 100 % at 1 h steps except a 70 % overnight step) at 4 °C. Following dehydration haustoria were embedded in Steedman’s wax for histochemistry or in LR White for immunofluorescence, immunogold labelling, ultrastructure imaging and Raman spectroscopy.

### Wax embedding and histochemistry of haustoria

Formaldehyde-fixed and dehydrated haustoria and prehaustoria (*R. minor* grown with *L. perenne* and without host, *O. vernus* grown with *A. elatius* and without host) were embedded in Steedman’s wax to facilitate staining with Mäule and Wiesner reagents, which results in no staining or very weak staining of resin-embedded samples. The wax was prepared by melting at 65 °C 800 g of polyethylene glycol 400 distearate (Sigma, 305413) and 100 g of 1-hexadecanol (Sigma, 258741) mixed and cooled to 37 °C. Up to ten haustoria per 1.5-mL Eppendorf tube were incubated in an oven (Fisherbrand) at 37 °C in 50 % of molten wax in EtOH for 2–4 h, 100 % wax overnight and 100 % wax for 4 h. The tubes were manually agitated for several seconds halfway through the first and last incubation step. Haustoria were then transferred to paper or silicon moulds filled with molten wax and left to set overnight at room temperature.

Wax-embedded samples were sectioned at ∼10 μm with disposable microtome blades (Edge-Rite^®^, Richard-Allan Scientific^®^) on a Leica RM2125RT microtome, floated on drops of dH_2_O on Vectabond™-treated (Vectabond™, Vector Labs) slides or polylysine slides (VWR, 631-0107) and allowed to flatten and dry at room temperature. Samples were subsequently warmed on a Leica HI1220 hotplate at 60 °C to increase section adhesion to slides followed by dewaxing in 100 % EtOH (2 × 10 min), 70 % EtOH–30 % dH_2_O (10 min), 50 % EtOH–50 % dH_2_O (10 min) and dH_2_O (10 min). Alternatively, sections were placed in 6-mm Petri dishes with ethanol, where they were dewaxed and rehydrated in a similar EtOH series.

Three histochemical dyes were used to visualize lignin: toluidine blue, Wiesner reagent and Mäule reagent. Toluidine blue is a polychromatic dye that stains lignin bright blue or green–blue while pectin-rich, non-lignified walls are stained purple (O'Brien *et al*., 1964). A 0.2 % w/v solution of toluidine blue in 1 % w/v borax (sodium tetraborate decahydrate) was applied for 1 min to dewaxed and rehydrated sections mounted on glass slides. Alternatively, dewaxed and rehydrated sections were carefully transferred with a small amount of dH_2_O using a Pasteur pipette and floated in a drop of the stain solution. The dye was washed away by three to five changes of dH_2_O. Sufficient washing was determined by observing in a stereoscope if there was any dye leaking out of the section 1 min after being placed in a small water droplet on a glass slide. Sections were mounted and observed in dH_2_O. Wiesner reagent stains polymeric lignin magenta red (Blaschek *et al*., 2020). Dewaxed but not rehydrated 10-µm sections were mounted in a solution of 1 % w/v phloroglucinol (Sigma, P3502) in 95 % EtOH mixed 5:1 with concentrated HCl immediately before observation and imaging. Additionally, whole fixed attached haustoria and metahaustoria were subject to staining in a Petri dish and observed in a stereomicroscope. Mäule reagent also stains lignin, whereby shades of red indicate syringyl-rich lignin and brown syringyl-poor lignin (Patten *et al*., 2007). Dewaxed and rehydrated 10-µm sections were stained with 1 % w/v aqueous potassium permanganate (KMnO_4_), rinsed in dH_2_O and incubated briefly in 3 % v/v HCl before a final rinse in dH_2_O. A couple of drops of concentrated ammonium hydroxide were subsequently added dropwise until strong contrast developed. The stained sections were mounted in glycerol and observed directly after staining.

### Resin embedding and sectioning of haustoria

London Resin White medium grade (LR White Resin, Agar Scientific, R1281) was introduced to dehydrated material in 1- to 2-h steps of 30, 50, 70 and 90 % resin in 100 % EtOH, 100 % resin for 1–2 h and an overnight step in 100 % resin on a rocker (Gyrorocker Stuart SSL3). Resin (100 %) was changed again, and samples were incubated for 1–3 h before transferring to gelatin capsules in which they were polymerized for 2 d at 60 °C. Resin-embedded samples were sectioned using a Reichert–Jung Ultracut ultramicrotome and a histodiamond knife (Diatome) at thicknesses specified for each imaging method.

### Fluorescent immunohistochemistry

London Resin White sections of 0.5 µm were mounted on dH_2_O droplets on Vectabond™-treated or polylysine slides and dried on a hot plate set to 40 °C for 30 min. Labelling was performed using rat monoclonal antibodies (mAbs) to cell wall glycans: LM11 to xylans (McCartney *et al*., 2005), JIM5 and LM19 or JIM7 and LM20 to de-esterified and esterified pectic homogalacturonan (HG), respectively (Clausen *et al*., 2003; Verhertbruggen *et al*., 2009), LM21 and LM22 to mannans (Marcus *et al*., 2010), LM25 to xyloglucans (Pedersen *et al*., 2012) and LM2 (Smallwood *et al*., 1996; Yates *et al*., 1996) to AGPs. Sections were incubated in 1 % w/v milk protein (dried skimmed milk) or bovine serum albumin (BSA, A4503, Sigma) in PBS for 30 min to eliminate non-specific binding, washed with PBS buffer (twice for 5 min per step) and incubated for 1.5 h in primary antibodies diluted 1:10 in blocking solution. Following three washes in PBS (5 min each), FITC-conjugated secondary antibodies (anti-rat, Sigma, F6258, or anti-mouse, Sigma, F0257) diluted 1:100 in the blocking solution were applied for 1.5 h. The sections were washed in several changes of PBS and poststained for 2 min with toluidine blue O (0.2 % w/v in 1 % w/v aqueous sodium tetraborate) to quench intense autofluorescence. Poststained and buffer-washed samples were mounted in glycerol-based antifade mounting reagent Citifluor AF2 (Agar Scientific) and imaged using an Olympus BX51 epifluorescence microscope with an X-Cite^®^ 120Q mercury lamp, Olympus XC10 digital camera and Cell B software.

### Electron microscopy and immunogold labelling

London Resin White sections of gold (∼90 nm) or silver (∼60 nm) interference colour were obtained on a Reichert–Jung Ultracut ultramicrotome and a Diatome histodiamond knife using part of the knife edge dedicated to ultrathin sections only. The 90-nm sections for ultrastructural observations were mounted on non-coated 200 square mesh Cu grids (TAAB) washed for 5 min in absolute acetone and air-dried. The 60-nm sections for immunogold labelling were mounted on formvar-coated nickel 2 × 1 mm slot grids (TAAB), incubated on droplets of freshly prepared ammonium chloride (0.13 g in 50 mL dH_2_O, 48.6 mm) for 15 min to block residual aldehydes from fixative and washed on three droplets of dH_2_O. To block non-specific antibody binding the grids were subsequently incubated in 1 % BSA in PBST (PBS, Agar Scientific, P5493) with 0.1 % v/v Tween^®^20 (Sigma, P1379). Grids were washed by placing them on drops of PBST (three changes, 5 min each). Grids were floated on 30-µL droplets of primary antibody JIM5 to de-esterified pectic HG (Verhertbruggen *et al*., 2009) diluted 1:10 in the blocking solution and mouse anti-MLG mAb (Meikle *et al*., 1991) diluted 1:100 for 2 h at room temperature in a plastic box with moist filter paper covered with a lid. After rinsing the grids on PBST droplets (five changes, 3 min each) 10 nm gold-conjugated goat anti-rat or goat anti-mouse IgG secondary antibodies (Ted Pella) diluted 1:75 in the blocking solution were applied. Sections were contrasted by floating the grids on drops of uranyl acetate (1 % w/v in dH_2_O, centrifuged for 2 min before use) covered by a light-opaque lid for 10 min, followed by five drops of dH_2_O for 1 min each. Reynolds lead citrate was filtered through a 0.22-µm syringe filter just before use and grids were floated for 3 min on drops in a glass Petri dish filled with sodium hydroxide pellets to absorb carbon dioxide and prevent carbonate precipitation. dH_2_O decarbonized by boiling and cooled down to room temperature was used for washing the lead citrate-stained grids to further reduce carbonate precipitates. Grids were air-dried for several hours before observations. For some samples heavy metal precipitates occurred at levels obscuring the observed features and imaging was repeated on non-contrasted sections. Contrasting, if applied, is indicated in the figure descriptions. Images were acquired digitally on a Zeiss Libra^®^ 120 (120 kV) microscope with a slow-scan CCD camera (Albert Tröndle Restlichtverstärkersysteme) and on a Hitachi H7000 transmission electron microscopewith a Hamamatsu 1 K digital camera.

### Raman spectroscopy

Raman spectroscopy was performed using a Renishaw^®^ InVia Raman spectrometer coupled to a Leica microscope equipped with a motorized XYZ stage. A green laser (514 nm) was used to excite the samples, according to the manufacturer’s instructions. Spectral measurements at fixed wavelength were investigated for a range of published bands reported in the literature as characteristic of lignin phenolics and the phenolic and aliphatic domains of suberin (Prinsloo *et al*., 2004; Busch *et al*., 2010; Schmidt *et al*., 2010). The results of these preliminary investigations showed that distinctive Raman-shifted emission peaks were obtained only for the lignin-related region lying between wavenumbers 1300 and 1700 cm^−1^, and all subsequent investigations were made using this region. Haustoria of *R. minor* attached to *A. elatius* embedded in LR White medium resin as described were sectioned at 1 μm, providing a good signal/autofluorescence ratio. The sections were mounted on non-coated glass slides washed with water with household detergent and rinsed with distilled water and 100 % EtOH. Mapping was carried out at ×50 magnification, 1-μm resolution and 100 % power with cosmic ray removal, and 10-s exposure. Data were analysed in MATLAB R2012 (Mathworks) using principal component analysis (PCA) and multivariate curve resolution (MCR) tools from the PLS Toolbox (Eigenvector Research Inc.) after normalization and smoothing (Savitzky–Golay). Additionally, data were mean-centred for PCA but not for MCR. PCA was highly useful in assessing the number of components applied in MCR analysis as well as detecting correlation (both positive and negative) between different parts of the spectrum. Loadings from MCR corresponded more tightly with spectra of individual biochemical components and were therefore more chemically significant.

The number of haustoria analysed by each method is indicated in Table 1. The haustoria were collected from different individuals except where 20 haustoria are indicated as these came from 10–12 individuals.

**Table 1.** Composition of LID. The number of haustoria with positive histological or immunofluorescence staining is stated with species indicated as *R*, *Rhinanthus minor*; *O*, *Odontites vernus* or expressed as −, present in none of the analysed haustoria; +, present in all analysed haustoria; (+) weak staining in all analysed haustoria. Inf, infective haustoria; Pre, prehaustoria.

Detection method		Specificity	Interfacial cells		LID		Xylem and host endodermis	Number of analysed haustoria			
			Inf	Pre	Pre	Inf	Inf and host	Inf–*R*	Pre–*R*	Inf–*O*	Pre–*O*
Monoclonal antibodies	LM2	AGP glycan	+	+	+	+	−	20	10	5	5
	LM19 and JIM5	De-esterified HG	+	+	−	−	−	20	10	5	5
	LM20 and JIM7	Esterified HG	+	+	−	−	−	20	10	5	5
	LM15	Xyloglucan	+	+	+	3*R*/1*O*	−	15	15	3	3
	LM25	Xyloglucan	+	+	+	3*R*/1*O*	−	15	15	3	3
	LM11	Xylan	−	−	−	−	+	15	15	3	3
	LM21/22	Mannan	−	−	−	−	−	15	15	3	3
	Anti-MLG	MLG	−	−	−	−	−	15	15	3	3
Wiesner reagent		Lignin	3*R*/2*O*	3*R*/2*O*	+	+	(+)	10	10	5	3
Mäule reagent		Lignin	(+)	(+)	+	+	(+)	5	5	3	3
Raman 1600 wavenumbers cm^−1^	Blunt peak	Lignolic substance	+	+	+	+	+	3	3	0	0
	Double steep peak	Xylem lignin	−	−	+	+	+				

## RESULTS

The 4/0 °C stratification regime resulted in more abundant germination of *R. minor* and *O. vernus* seeds than stratification in the refrigerator (mean 42 % for *R. minor* at 4/0 °C, mean 20 % for *R. minor* at 4 °C, mean 49 % for *O. vernus* at 4/0 °C, mean 19 % for *O. vernus* at 4 °C). On average 99 % of *A. elatius* ssp. *bulbosum* seeds germinated after 3 d. Germination of *L. perenne* in trays was not quantified but ∼20 % of the seeds appeared to have germinated.

**Fig. 1.**
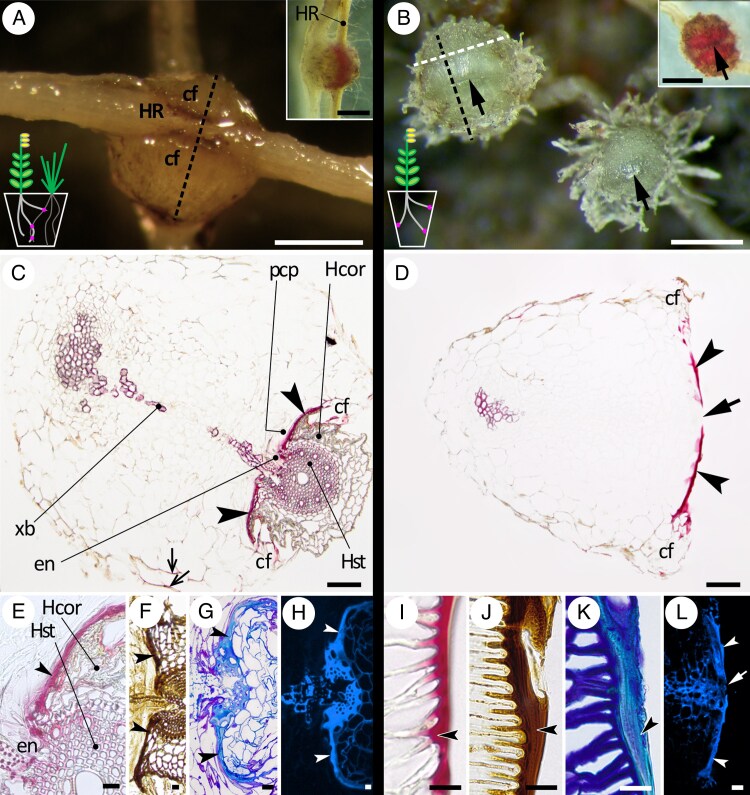
Morphology and lignin deposition in infective haustoria and prehaustoria of *R. minor*. (A, C, E–H) Infective haustoria of *R. minor*. (B, D, I–L) Prehaustoria of *R. minor*. (A) A mature infective haustorium of *R. minor* attached to an *L. perenne* root. (Inset) Wiesner reagent staining the interface between a haustorium and *L. perenne* root. (B) Prehaustoria with a flat distal surface featuring a slit where a host root would normally be found in a successful graft. (Inset) Distal surface of a prehaustorium stained with Wiesner reagent. (C, D) Wiesner reagent staining of an *R. minor* haustorium attached to *L. perenne* and an *R. minor* prehaustorium, respectively. (E–H) Lignin detection at haustorial interfaces with host roots and (I–L) in the corresponding region of prehaustoria, respectively. (E, I) Wiesner reagent, (F, J) Mäule reagent, (G, K) Toluidine Blue and (H, L) autofluorescence in the blue channel. Hosts are *L. perenne* (A, C, E, F) and *A. elatius* (G, H). (C, D, E–H, L) Sections collected in the direction indicated by the black dashed lines in images (A) and (B), i.e. perpendicular to the host root and the corresponding prehaustorial slit, respectively. (I, J, K) Sections collected as indicated by the white dashed line in (B), i.e. parallel to the slit, in the lateral region where the clasping folds partly cover the LID. (G, H) LR White-embedded samples. (A–F and I–L) Steedman’s wax-embedded samples. Scale bars: (A, B and insets) = 500 µm, (C, D) = 100 µm, (E–L) = 20 µm. HR, host root; cf, haustorial clasping fold; arrow with filled arrowhead, slit in the metahaustorial adhering surface; arrow with line arrowhead, lignified walls of haustorial parenchyma; concave kite arrowhead, LID; xb, xylem bridge; en, endophyte; pcp, parasite’s contact parenchyma; Hst, host root stele; Hcor, host root cortex.

**Fig. 2.**
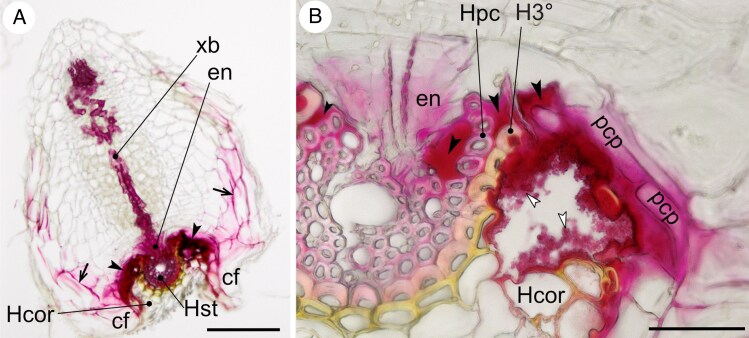
Wiesner reagent staining of lignin in a haustorial connection between *O. vernus* and *A. elatius* ssp. *bulbosum*. (A) Section through a whole haustorium attached to a host root. The reagent stains not only the vasculature of the xylem bridge but also extends far into the parenchymatous tissues of the haustorium. Host stele is also stained. The endophyte tip has penetrated the host stele, and its cells have differentiated into xylem. (B) Detail of the interfacial region of the same haustorium as in (A). Strongly stained infLID tightly fills the spaces between haustorial and host cells. Parasite contact parenchyma has partly grown between the tertiary endodermis and pericycle, separating them from each other. Black concave kite arrowhead, LID interface; white concave kite arrowhead, infLID with granular structure surrounding a rupture inflicted during preparation of haustorium; black arrow, lignified cell walls of haustorial parenchyma; xb, xylem bridge; en, endophyte; cf, clasping folds; Hst, host root stele; Hcor, host root cortex; pcp, parasite’s contact parenchyma; Hpc, host root pericycle; H3°, host root tertiary endodermis. Scale bars: (A) = 200 µm, (B) = 50 µm.

Infective haustoria (Fig. 1A) formed in all parasite–host pairings. Ten haustoria of *R. minor* and ten of *O. vernus* grown with *A. elatius* were collected from each round 7.6 cm pot and fixed. Remaining haustoria were not quantified. Prehaustoria developed on roots of *R. minor* and *O. vernus* grown with and without hosts and they all occurred on the outer surfaces of the root balls in contact with the container inner walls. Both parasite species produced two to five prehaustoria when grown with *A. elatius* in 7.6-mm pots, in addition to infective haustoria. *Rhinanthus minor* grown without hosts in cavity inserts produced on average 18 prehaustoria per insert. *O. vernus* grown without hosts in trays produced 13, 16 and 21 prehaustoria per tray (*O. vernus*) and *R. minor* produced 13, 14 and 17 prehaustoria per tray. Prehaustoria (Fig. 1B) were of similar size to host-attached haustoria. The distal part corresponding to where infective haustoria clasp around host roots was, however, appressed to the pot wall and had therefore developed a flat, smooth surface (Fig. 1B, D) instead of the penetrative peg, i.e. the endophyte (Fig. 1C), presumably in response to mechanostimulation. This contact surface was readily separated from the plastic pot wall without leaving any marks on it. As described below in more detail, it consisted of a layer of heterogeneous polyphenolic-rich extracellular deposit, bearing considerable ultrastructural and biochemical resemblance to substances found at the interface between infective haustoria and host tissues. We have named this substance lignin-rich interfacial deposit (LID). The abbreviation LID will henceforth be used throughout this manuscript to refer to the interfacial deposits found in both prehaustoria and infective haustoria, whereas preLID and infLID, respectively, will be used where a distinction is needed. All investigated aspects of LID composition, i.e. lignin and cell wall carbohydrate presence, are summarized in Table 1. LID stains positively for lignin with Wiesner (Fig. 1C, D, E, I) and Mäule (Fig .1F, J) reagents, and the lignin-like nature of the interfacial deposits is further supported by light blue staining with Toluidine Blue (Fig. 1G, K) and very strong autofluorescence in the UV channel (Fig. 1H, L). Localization of the staining to the adhering surfaces of prehaustoria corresponds to the interface with the host in infective haustoria. Furthermore, lignin staining extended into the haustorial contact parenchyma as well as parenchyma far away from the interface (Figs 1C and 2). At the interface infLID tightly fills the spaces between host and parasite cells.

**Fig. 3.**
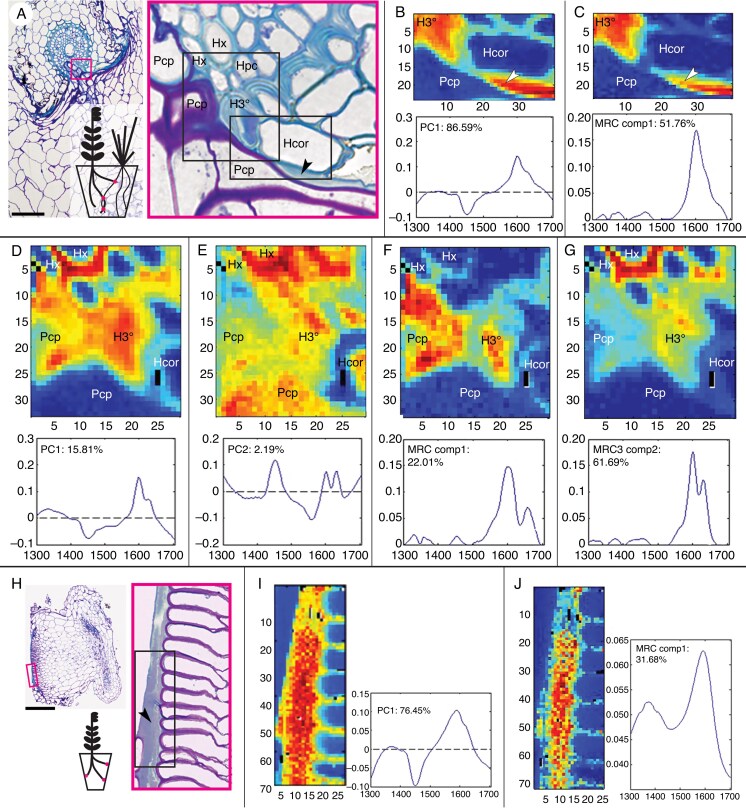
Raman spectroscopy of phenolic compounds in LID of *R. minor*. (A–G) *Rhinanthus minor* attached to *A. elatius* ssp. *bulbosum* and (H–J) *R. minor* prehaustorium. (A, H) Overviews with the mapped region locations indicated by magenta boxes. PCA (B, D, E, I) and MRC (C, F, G, J) results. The component and the percentage of total data variance described by it is indicated in each subpanel graph. (D, G) Cell walls of host protoxylem display a double peak at wavenumber 1600 cm^−1^, characteristic of lignin (red scores). (G) Same peak is represented at lower pixel intensity (yellow and orange scores) in the cell walls of lignified tertiary endodermis. (F) MCR analysis resolves the double peak from a single peak at wavenumber 1600 cm^−1^. Single-peak lignin-like substance is localized in the protoplast of the crushed endodermal cell (F), cell walls of the parasite contact parenchyma cells (F), infLID (C) and preLID (J). (E) A peak near wavenumber 1450 cm^−1^ indicates background signal from cell protoplasts. The colour scale indicates pixel intensity for the imaged component (red, high; blue, low). Open kite arrowheads, LID; pcp, parasite’s contact parenchyma; Hcor, host cortex; Hx, host root protoxylem; H3°, host root tertiary endodermis. Scale bars: (A) = 100 µm; (H) = 200 µm. Map pixel size: 1 × 1 µm.

Raman spectroscopy results support the lignin-like nature of LID (Fig. 3). Both PCA and MCR deconvolution analyses identify a peak of wavenumber ∼1600 cm^−1^ characteristic of polyphenolics in xylem vessels and in LID. MCR additionally reveals a difference in curve topography between LID (Fig. 3C, F, J) and xylem wall-associated components (Fig. 3G). Namely, LID lignin is represented by a single broad peak whereas a steeper curve with a double peak is characteristic of xylem-localized lignin (Schmidt *et al*., 2010). The LID peak is also confined to substances filling the lumens of grass host tertiary endodermis cells compressed by the intrusive haustorium (Fig. 3F).

**Fig. 4.**
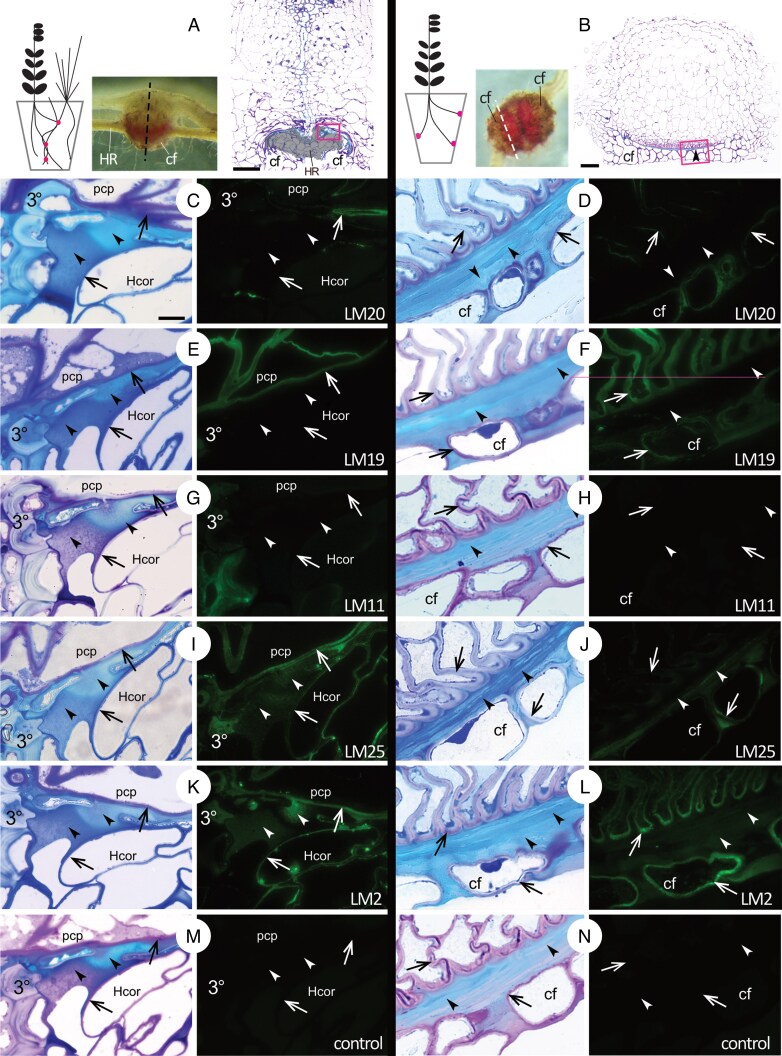
Immunofluorescence detection of cell wall carbohydrate epitopes in the LID. (A, B) Overview panels showing where the sections were collected in haustoria and prehaustoria, respectively, and which section regions are featured in (C–N). In (A) the greyed-out area of the section represents the host root. Magenta boxes show approximate locations imaged in (C–N). (C, E, G, I, K, M) InfLID at the interface between *R. minor* and compatible grass host *A. elatius* ssp. *bulbosum* in 500-nm sections taken from the same haustorium. (D, F, H, J, L, N) *Rhinanthus minor* preLID in 500-nm sections collected at the lateral part of prehaustorial face, where LID is found abundantly under a non-functional clasping fold. cf, clasping fold; HR, host root; pcp, parasite contact parenchyma; Hcor, host cortex parenchyma; 3°, host tertiary endodermis. Arrowheads and arrows indicate corresponding regions of LID and cell walls; respectively, in bright-field images of Toluidine Blue staining and fluorescence micrographs. Antibodies: LM 20, esterified pectic homogalacturonan; LM19, de-esterified pectic homogalacturonan; LM11, xylan; LM25, xyloglucan; LM2, AGP glycan. Scale bars: (A, B) = 100 µm, (C) = 10 µm and applies also to (D–N). Declaration of image manipulation: (D, F, H, J, L, N – bright-field and immunofluorescence) are stacks of several images due to the plastic sections not adhering evenly. Contrast was adjusted across the whole of image (I).

No pectin (Fig. 4C–F) or xylan (Fig. 4G, H) was detected in LID by immunofluorescence. This suggests lack of a carbohydrate scaffold typical of either dicot or monocot cell walls in the LID. However, xylans were present in the lignified cell walls of the haustorial xylem bridge and in grass host cells (Fig. 4G). Pectic HGs, particularly de-esterified HGs as detected by LM19, were abundant in the cell walls of the haustorium and provided a clear distinction between the cells of the parasite and the graminoid host at the interfacial region. Two classes of cell wall polysaccharides are detected in the LID, namely xyloglucans and AGP glycans (Fig. 4I–L). The antibody LM2 against AGPs labelled the LID in all examined haustoria and prehaustoria. AGP epitopes were also detected in haustorial interfacial cell walls and host cell walls (Fig. 4K, L) as well as in the cell walls, and the content of some crushed host cells at the very interface with the parasite (personal observation). Xyloglucan was always labelled with LM25 in preLID but infLID contained xyloglucan only in 3 of 15 *R. minor* haustoria and 1 of 3 *O. vernus* haustoria. Heterogeneity of Toluidine Blue staining in the LID corresponds to that of AGP and xyloglucan detection. In prehaustoria xyloglucan is confined to or detected with higher intensity in certain layers of preLID (Fig.4J). In TEM the layers revealed by toluidine blue staining and immunofluorescence display different electron opacity, even without osmium postfixation. In attached haustoria the xyloglucan located to infLID if it displayed a somewhat grainy texture (Fig. 4K), but not when it was smooth.

**Fig. 5.**
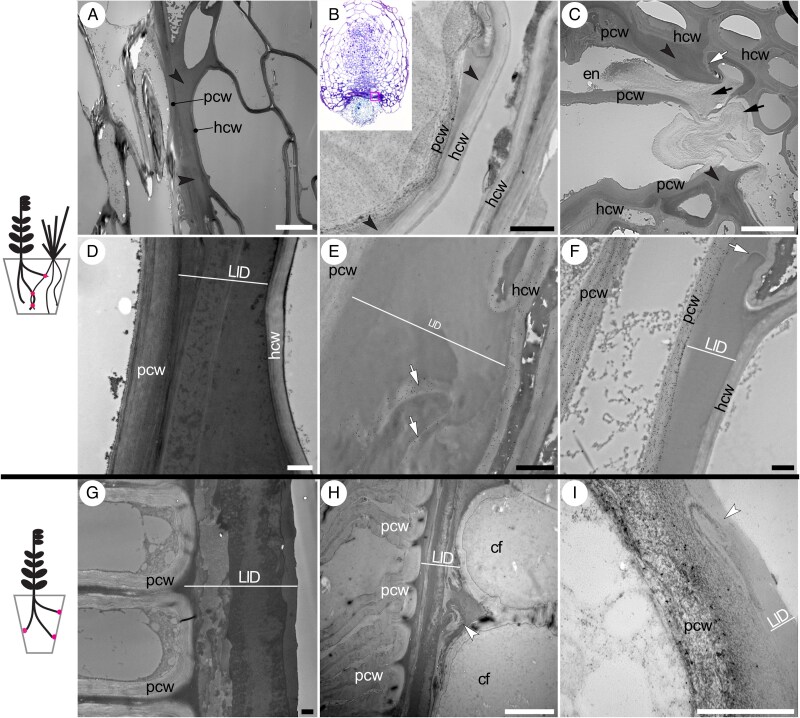
Ultrastructure of LID. (A–F) LID in infective haustoria (infLID) and (G–I) in prehaustoria (preLID). (A) infLID tightly fills the space between the parasite *R. minor* (left) and host *A. elatius* (right) cells at the lateral parts of the interface. (B) infLID of an *R. minor* haustorium that has begun penetration into the host *A. elatius* tissues, before the penetrative peg (endophyte) has formed. The walls of the parasite are loosely fibrillar, forming protrusions towards the host walls. (C) *R. minor* haustorial endophyte tip with disintegrating end cell walls. infLID tightly fills all the spaces between the parasite and host cells. (D) Layered ultrastructure of infLID at the lateral interface between *R. minor* and *A. elatius*, with blotches of higher electron opacity and no visible fibrillar structure. (E) Immunogold labelling of MLG in interrupted host *A. elatius* cell walls embedded in infLID at the interface with *R. minor*. (F) Immunogold labelling of homogalacturonan with mAb JIM5 in the walls of *R. minus* contact parenchyma. No labelling is present in infLID or *A. elatius* cell walls (G) *R. minor* preLID in a region that was in direct contact with the pot surface. (H) *R. minor* preLID in the lateral region under a clasping fold. Layered ultrastructure and tight association with the haustorial cell walls are apparent. (I) JIM5 immunogold labelling of homogalacturonan pectin in contact parenchyma walls in *O. vernus* prehaustoria. Black arrowheads, LID; white arrowheads, areas of LID with malleable appearance; black arrows, disintegrating cell walls of haustorial endophyte; white arrows, interrupted delamellated host cell walls embedded in LID; pcw, parasite cell wall; hcw, host cell wall; cf, clasping fold; en, endophyte. The haustorium shown in (A) was osmicated and stained on-section with uranyl acetate and lead citrate. Samples shown in (B–I) were not osmicated and are unstained. White scale bars = 10 µm, black scale bars = 1 µm.

The LID is always very tightly associated with the cell walls of the parasite. We observed no instances in which it mechanically separated from the fibrillar fraction of the parasite’s cell walls and it was always anchored between the distal ends of parasite contact cells (Figs 1I, J and 5G). The thickness of the LID layer varies considerably between various areas of the interface depending on how its topography was shaped by entry into host tissues. The most abundant accumulation is typically seen as wedges separating host root cortex, endodermis and pericycle from each other (Figs 2B and 4C, E, G, I, K, M). A very thin and homogeneous LID layer seen only at ultrastructural level is characteristic of the central interface where the endophyte meets host cells in the stele (Fig. 5C). It is also seen at early developmental stages when the haustorium is beginning to penetrate the host cortex (Fig. 5B). It is in the latter two cases that haustorial and host cell walls come very close to each other but are nevertheless mostly interspaced by a thin layer of LID. In mature haustorial connections the ultrastructure of infLID ranges from smooth, typically when only a thin layer is present (Fig. 5F), to coarsely mottled or layered (Fig. 5D). In some cases, the infLID layers appear to correspond to sequentially disintegrated host cells. Debris of host cell walls is commonly found embedded in the infLID (Fig. 5C, E, F) and bacteria were occasionally also observed. In *R. minor* preLID, a distinct layered structure is common. A range of features suggesting a temporary liquid, malleable state and intermixing of sublayers can be also observed in the form of ‘swirls’ (Fig. 5H, I). While LID texture shows considerable variation, a clear fibrillar component is consistently missing, making LID easily distinguishable from the neighbouring cellulose-based cell walls.

## DISCUSSION

### The origin of lignin-rich interfacial deposits

Cell wall remodelling undoubtedly plays a major role in haustorium development (Kokla and Melnyk, 2018; Brun *et al*., 2021) and lignification of the xylem bridge is an inherent feature of haustorial vascularization. In the context of antagonistic interactions between plants and pathogens, lignin is unequivocally associated with host defence mechanisms and any such substance found at interfaces between parasitic plant haustoria and their host plants is typically also assumed to be a product of the host. Moreover, host lignin is a source of HIFs and host lignin composition can affect haustorium induction (Cui *et al*., 2018). Further, the results presented in this paper suggest that at least a proportion of the interfacial lignolic substances may originate from the parasite.

The most direct proof of lignin synthesis by the parasite is the ability of prehaustorial distal cells to secrete LID in the absence of a host and in contact with a biologically inert object, i.e. the plastic wall of the pot. That prehaustoria can mimic LID secretion characteristic of infective haustoria but independently of any specific biochemical cues from the host implies that its production is a generic and evolutionarily primitive process unrelated to host recognition.

Architectural similarities of infLID and preLID include the same classes of constituent cell wall polymers, i.e. lignin-like substances, AGP glycans and xyloglucans. The graminoid-specific MLG epitopes were detected only in the remnants of host cell walls trapped in the infLID but never in the surrounding infLID itself. Xyloglucans are abundant in dicot cell walls but occur only in low amounts in graminoid cell walls (Harris, 2005; Park and Cosgrove, 2015). They were, however, consistently detected in the grass host root in this study. A xyloglucan component of LID provides in this case only a limited clue that the deposits are synthesized by the dicotyledonous parasite.

In typical lignification responses triggered by wounding or parasitism lignin incrusts the host cell walls, cell wall appositions known as papillae, and middle lamellae (Vance *et al*., 1980; Nicholson and Hammerschmidt, 1992; Rümer *et al*., 2007; Mutinda *et al*., 2023). Therefore, the abundant accumulation of extracellular lignin-like substance seen in this study does not match a typical lignin-based histological defence response. Furthermore, host xylem penetration in the central region of the interface was not hindered even when the lignin deposition was abundant at the lateral regions. A thin interfacial lignified layer is present also in other haustorial grafts to compatible hosts, as seen for *Melampyrum arvense*, *Rhinanthus alectorolophus* and *O. vernus* ssp. *serotinus* connected to several different native and non-native hosts (Knotková *et al*., 2024), *Thesium humifusum* (Santalaceae) (Renaudin and Cheguillaume, 1977), *Striga hermonthica* and its wheat and sorghum hosts (Vasey *et al*., 2005)), as well as *S. hermonthica* and a compatible rice cultivar (Mutuku *et al*., 2019). In the latter study strong biochemical and molecular evidence for lignin accumulation in the non-host cultivar is presented. However, the accompanying micrograph of phloroglucinol staining shows somewhat stronger staining only at the very central part of the interface with the resistant cultivar while staining is equally strong at the lateral parts of the interface with both cultivars. In a recent study investigating sorghum’s resistance to *Striga* (Mutinda *et al*., 2023), phloroglucinol stained strongly the entire stele of genotypes displaying mechanical barrier resistance and only very weakly in a genotype employing a hypersensitive response at the interface as a defence mechanism. Unsurprisingly, upregulation of genes related to lignin biosynthesis was lower in the latter host genotype while, notably, the interfacial zone stained very strongly. Altogether these findings show that various lignification processes occur at and near the interfaces between haustoria and host tissues but not all of them result in successful host resistance. LID may therefore occur parallel to defence-related lignification. An increasing number of studies mark lignification and upregulation of lignin metabolism genes in the haustoria in addition to that in the host (Sun *et al*., 2018; Vogel *et al*., 2018; Brun *et al*., 2023; Cui *et al*., 2025; Leman *et al*., 2025). This opens new prospects for building on the histological evidence for lignin’s parasitic origin presented in this study. Distinguishing between upregulation related to xylem bridge formation and interface-localized lignification processes would be of particular interest.

### Composition and possible deposition mechanisms

Strong Wiesner reaction in the LID points to the presence of polymeric lignin and cannot be explained by other phenolic compounds such as ester-linked phenolic acids. The reaction, which has been traditionally used to detect lignin (Clifford, 1974), was recently confirmed to be specific to coniferaldehyde residues incorporated at the ends of and within lignin polymers (Blaschek *et al*., 2020). Furthermore, brown rather than red staining with Mäule reagent points to low content of syringyl units (Patten *et al*., 2007), something that has previously been ascribed to ‘defence lignin’ during wounding as opposed to the ‘developmental’ SG lignin of xylem (Hawkins and Boudet, 2003). The Raman curves for LID have broad peaks at wavelengths specific for lignin. This broader topography is also seen for substances filling crushed host cells, in contrast with the steep double peaks observed for lignified xylem walls. The differences may be related to monolignol composition, whereby the double peak is due to the S and G units (Larsen and Barsberg, 2010), reinforcing the results of Mäule staining. Crosslinking to the apparently less organized carbohydrate scaffold or density can also play a role. Since even preLID stains brown and displays the blunt Raman peak, the lignin formed by the parasite appears to bear more structural similarity with the lignins produced during defence responses than those found in vascular tissues.

LID raises questions about extramural deposition mechanisms of cell wall components. Lignin is typically deposited *in muro* within a pre-existing carbohydrate scaffold (Lewis and Yamamoto, 1990; Donaldson, 2001) and its supramolecular structure depends on the carbohydrate matrix type (Donaldson, 1994). In secondary walls with highly organized carbohydrate structure, it occurs in distinct layers (or lamellae) corresponding to the direction of cellulose microfibrils and parallel to the cell surface (Lamport and Várnai, 2013) while in middle lamellae and primary walls, with lower levels of carbohydrate network organization, lignification starts at multiple initiation sites from which it spreads and merges in all directions (Donaldson, 1994). While the mature preLID sometimes appears as a coarsely layered structure coinciding with differences in electron density, no fibrillar component characteristic of pectin or cellulose is present in preLID or infLID. The layering may reflect repeated cycles of drying out and rehydration at the pot surface during formation of the prehaustoria. It may also reflect the repeated intrusion attempts that have been observed for some parasite species as part of their normal penetration mode or in response to the host being particularly difficult to penetrate (Kuijt, 1965). The apparent lack of pectin is in contrast with the cementing secretions of *Cuscuta* (Vaughn, 2002) and three species of Orobanchaceae (Neumann *et al*., 1999), where HG epitopes were detected. Lack of a highly organized, crystalline cellulosic component is further confirmed by the fact that LID is not birefringent (A. Pielach pers. obs.), a property resulting from the orientation order and crystallinity of cellulose in the walls (Pate and Gunning, 1972; Yu *et al*., 2005; Nakagawa *et al*., 2012). Moreover, xylan, which is a hemicellulose characteristic of lignified cell walls such as those of xylem, is absent from LID. Immunodetection points instead to a high content of AGP moieties and, in some haustoria, xyloglucans, which have somehow made their way across the thick cellulosic walls of haustorial contact cells. Xyloglucans have been demonstrated to be exuded by plant roots and to promote soil particle aggregation (Galloway *et al*., 2018) but the mechanisms of their release are not known. Furthermore, xyloglucan metabolism is a feature of haustorial development in the shoot parasite *Cuscuta* (Olsen *et al*., 2015). AGP glycan moieties are believed to be large and relatively immobile (Lamport and Várnai, 2013). Nevertheless, their ability to modify the properties of cell wall polymers by plasticizing pectins through decreasing their cross-linking (Lamport and Kieliszewski, 2005; Lamport *et al*., 2006) and affecting the action of xyloglucan transglycosylases (Takeda and Fry, 2004) has been hypothesized to enable AGPs to facilitate their own progress across the wall by modifying the physical obstacles within it (Lamport and Kieliszewski, 2005; Knox, 2006). They could also play an important role in plasticizing the LID itself during the establishment of the haustorium. Active secretion of extracellular lignin and lignin-like substances is to our best knowledge documented only in culture cells where it happens to be linked to pathogenesis markers. *Zinnia* (Helianthaceae) xylem cell precursor cultures were inhibited by chitosan and fungal elicitors from differentiation into immature tracheary elements and instead secreted an extracellular lignin-like substance (Takeuchi *et al*., 2013), and cell cultures of *Pinus taedea* (Pinaceae) (Nose *et al*., 1995) and *Picea abies* (Pinaceae) (Brunow *et al*., 1993) were found to exude lignin of different composition to wall-bound lignin.

### Possible functions of lignin-rich interfacial deposits

Overall, the architecture of LID suggests deposition mechanisms that differ from those involved in lignification of vessels and fibres. Lack of directional fibrillar scaffold and high content of potentially plasticizing carbohydrates (AGPs) may render LID a malleable material, something that is supported by our observation of swirls at the ultrastructural level. This plasticity would be a beneficial property for sealing the point of entry to prevent solute leak and embolism and thereby ensure the integrity and correct functioning of the haustorial connection. On the other hand, interception of microbes, disintegrating host cells and neutralization of toxic metabolites released by the latter are possible functions. The presence of phenolics throughout allows rapid crosslinking (Cooper-Driver, 2001), which might reinforce the haustorial graft and make it withstand the mechanical forces that are applied during the establishment phase and lead to the outer host cortex being crushed. This anchoring function is supported by the fact that LID is observed early on in haustorial development, before the endophyte appears.

In conclusion, our results suggest that interfacial lignin deposition is not attributed solely to the host, calling for functional and molecular investigations and a possible reinterpretation of its function in the parasitic plant–host interactions. The potential for lignin deposition to benefit the parasite and its potential roles in parasitism should be investigated in parallel to the host-centric functions such as mechanical barriers to the haustorium and as a source of HIFs. The ability of *R. minor* and *O. vernus* to synthesize interfacial lignin is thus a new important piece of the puzzle, paving the way for further interdisciplinary studies where molecular, biochemical, mechanical and functional aspects are of interest.
